# Investigating Outcomes of a Family Strengthening Intervention for Resettled Somali Bantu and Bhutanese Refugees: An Explanatory Sequential Mixed Methods Study

**DOI:** 10.3390/ijerph191912415

**Published:** 2022-09-29

**Authors:** Sarah Elizabeth Neville, Kira DiClemente-Bosco, Lila K. Chamlagai, Mary Bunn, Jordan Freeman, Jenna M. Berent, Bhuwan Gautam, Abdirahman Abdi, Theresa S. Betancourt

**Affiliations:** 1 Intenational Health Institute, School of Public Health, Brown University, Providence, RI 02903, USA; 2 Center for Dissemination and Implementation Science, Department of Medical Social Sciences, Feinberg School of Medicine, Northwestern University, Chicago, IL 60611, USA; 3 Brown Mindfulness Center, Behavioral Health and Social Science Department, School of Public Health, Brown University, Providence, RI 02903, USA; 4 Department of Psychiatry, University of Illinois at Chicago, Chicago, IL 60612, USA; 5 Bill & Melinda Gates Institute for Population and Reproductive Health, Bloomberg School of Public Health, Johns Hopkins University, Baltimore, MD 21205, USA; 6 Research Program on Children in Adversity, School of Social Work, Boston College, Chestnut Hill, MA 02467, USA; 7 College of Medicine, Pennsylvania State University, Hershey, PA 17033, USA; 8 Shanbaro Community Association, Chelsea, MA 02150, USA

**Keywords:** prevention, child mental health, parenting, community-based participatory research, home visiting

## Abstract

Pre- and post-migration stressors can put resettled refugee children at risk of poor mental health outcomes. The Family Strengthening Intervention for Refugees (FSI-R) is a peer-delivered preventative home visiting program for resettled refugees that aims to draw upon families’ strengths to foster improved family communication, positive parenting, and caregiver-child relationships, with the ultimate goal of reducing children’s risk of mental health problems. Using an explanatory sequential mixed methods design, this study draws upon qualitative interviews with caregivers (n = 19) and children (n = 17) who participated in a pilot study of the FSI-R intervention in New England, as well as interventionists (n = 4), to unpack quantitative findings on mental health and family functioning from a randomized pilot study (n = 80 families). Most patterns observed in the quantitative data as published in the pilot trial were triangulated by qualitative data. Bhutanese caregivers and children noted that children were less shy or scared to speak up after participating in the FSI-R. Somali Bantu families spoke less about child mental health and underscored feasibility challenges like language barriers between caregivers and children. Interventionists suggested that families with higher levels of education were more open to implementing behavior change. In both groups, families appreciated the intervention and found it to be feasible and acceptable, but also desired additional help in addressing broader family and community needs such as jobs and literacy programs.

## 1. Introduction

The global population of refugees—individuals who are forced to leave their native country and cross international borders searching for safety from war, violence, persecution, and other natural disasters—has doubled in the last decade as a result of ongoing global conflict, up to approximately 26 million [1]. Perhaps most remarkable is the extent to which refugee communities overwhelmingly demonstrate profound strength and resilience in the face of such tremendous adversity. Research with diverse refugee communities has identified numerous individual, family and community-level resources (faith, family and community relationships, cultural values and practices) which refugees rely on to overcome difficulty and support adjustment and wellbeing [2,3].

At the same time, research also indicates that exposure to violence, trauma, and loss experienced in country of origin and during migration increases risks for common mental disorders (e.g., depression, anxiety, post-traumatic stress). Resettlement to the United States and other host countries introduces additional post-migration stressors and living difficulties (e.g., economic pressures, legal status, education and health care access) which often exacerbate risks to mental health. Several systematic reviews have identified high rates of depression, anxiety, and post-traumatic stress disorder (PTSD) among refugee adults and children [4,5,6,7]. Bogic et al., for example, found that resettled refugees in Western countries were 14 times more likely to be depressed and 15 times more likely to experience PTSD compared to the general population [4]. Similar disparities have been identified in refugee children and adolescents [6].

Forced migration can also separate families from extended kin, change family roles, and upend family configurations, potentially resulting in poor family functioning and child mental health consequences [8]. Traumas and stressors experienced across the migration continuum may also adversely impact the entire family system and can result in decreased family cohesion, reliance on harsh or ineffective parenting strategies, negative parent–child interactions, and increased risk of family conflict [9].

These shifting family dynamics, in turn, can further increase risk of mental health and psychosocial problems for children. For example, resettled refugee children sometimes adopt new roles to help their family meet their daily needs, such as interpreting or functioning as a cultural mediator for their parents. While adaptive in nature, this role expansion can complicate family dynamics and lead to anxiety and stress in children who may be exposed to sensitive or distressing information or pressures inappropriate for their age [10]. In addition to refugee children’s own direct experiences of trauma and stress, parental exposure to past trauma and ongoing adversity can affect parenting. For example, it may reduce their daily functioning or result in emotional dysregulation or emotional distance from children, dynamics which increase children’s risk of mental health problems [11,12]. Parenting can be complicated by resettlement, as caregivers find themselves in a new culture in which parenting practices from their country of origin may be at odds with parenting norms expected in their new environment, with limited support from extended kin or friends to assist with emotional needs or childcare responsibilities [13]. Parental isolation can contribute to poor parental mental health and resultant unhealthy parenting patterns contributing to increased family and parent–child discord. Such vulnerabilities create a family context that is challenging for children at a time when they most need assistance and support to navigate a new culture and context as resettling refugees [14].

Yet, it is important to also highlight the strengths and perseverance that refugee families and communities commonly exhibit, and which can be leveraged to support wellbeing and adjustment. The family is an important resource for refugee families and functions as a pivotal source of support for coping with past and ongoing challenges [15]. A study with Iraqi refugee families, for example, found that close family relationships, cultural practices in the home and a renewed focus on their future were critical ingredients in making meaning of resettlement related stress and adversity [16]. Indeed, family relationships are crucial to children’s developing sense of culture and identity. This is particularly true for many refugee families, where identity is often strongly grounded in relation to one’s family and community [17]. Moreover, many refugees rely more strongly on their families for support in the post-migration period, as migration can interrupt their previous social support networks. Positive social support from the family has been found to mediate the negative mental health outcomes experienced by refugee adults [18] and has been associated with better psychosocial functioning in refugee children [19]. For refugee children and adolescents, family-level factors including engaged parenting and family cohesion have been found to be important to fostering long-term resilience [20]. Thus, strengthening family support, functioning, and resilience may be a critical approach for mitigating the effects of trauma and stress in families and promoting child mental health [21,22,23].

### 1.1. Resettled Bhutanese and Somali Bantu Communities in New England

New England’s resettled Bhutanese community is an ethnically Nepali refugee population who had been residing in Bhutan while retaining their Nepali language and customs for several generations. In the 1980s, the Kingdom of Bhutan embarked upon a campaign of “Bhutanization” that resulted in many human rights abuses, including death threats and torture, towards the Lhotshampsa population (ethnically Nepali Bhutanese) and their displacement from the country. They subsequently lived in refugee camps in eastern Nepal for over 20 years [14]. From 2007 to date, over 110,000 Bhutanese refugees have been resettled to the United States [24].

Resettled Bhutanese communities demonstrate strong resilience in the face of adversities, including a strong “culture of helping” in which families and communities proactively provide social support to individuals facing psychosocial challenges [25,26]. However, they also demonstrate high levels of mental health problems. Literature has found that experiencing torture was associated with mental health problems for Bhutanese not only while living in camps in Nepal [27], but also three decades later in the United States [28]. Research has also found that having less education and being illiterate in Nepali is linked to psychological distress, depression, and post-traumatic stress in resettled Bhutanese [29,30]. Most alarmingly, resettled Bhutanese have an increased risk of suicidal ideation, with rates of suicide being nearly double the US average in 2013 [31,32]. This increased risk of adult suicide has been associated with perceiving oneself as a burden and a lack of a sense of belonging, which are in turn linked to struggles obtaining employment, poor health, and family conflict [33]. Other risk factors for suicide include unemployment, substance use, family discord, isolation and family separation [34,35]. One study of resettled Bhutanese found that three-quarters believed other people would look down on them if they pursued mental health counseling, and this was more common amongst participants without a secondary education [36].

A large community of Somali Bantu refugee families have resettled in the New England area as well. “Somali Bantu” is an imperfect umbrella term for ethnic minorities in Somalia who were both indigenous to the country and those forcibly brought as enslaved people from other African regions starting in the nineteenth century [37]. The Somali Bantu mostly settled in farming communities along the Jubba and Shabelle rivers [37]. Following the collapse of the Somali government in 1991, much of the Somali Bantu population escaped the violence and fled to refugee camps in Dadaab and Kakuma, Kenya. Identifying the Somali Bantu as a persecuted minority group in 1999, the US government resettled about 10,000 Somali Bantu refugees between 2004 and 2006 [38].

Somali Bantu refugees have demonstrated high rates of problems often associated with trauma exposure and post-resettlement challenges such as depression, PTSD, anxiety and a high involvement of youth in the juvenile justice system [39,40]. Research with a mix of Somali-majority and Somali Bantu youth found that cumulative trauma was a risk factor for depression and PTSD, and discrimination was also linked to depression [40]. Indeed, youth within this community face the challenges of negotiating intersectionality of marginalized identities in the United States, including being black, Somali, and Muslim. In the context of such obstacles, schools have reported some Somali Bantu students struggle with behavioral problems [41]. Nevertheless, the community draws resilience and strength from retaining a strong cultural identities, commitment to religion and spirituality, and a sense of community and interdependence with other Somali Bantu that crosses distance and geographic boundaries [2]. While Somali Bantu youth have low rates of service utilization and evidence exists of stigma towards mental health problems, some Somali Bantu youth and adults have said they feel they can turn to religious leaders and the Quran in times of psychosocial need [42,43,44].

### 1.2. The Family Strengthening Intervention for Refugees (FSI-R)

To respond to the significant mental health needs and strengthen adaptive capacities in resettled refugee communities in New England, researchers and community partners began using community based participatory research (CBPR) approaches in 2004 to assess and collaboratively develop family based programs for refugee children and adolescents. Following CBPR best practices, researchers partnered with existing community groups, first in the Somali Bantu and then in the Bhutanese community, with intentionality towards deconstructing power dynamics. Towards this end, community members were involved in each stage of the intervention development and delivery process [45]. Involvement included the use of community advisory boards (CABs); extensive measurement tailoring within each community; hiring and training community members as research assistants and interventionists; and collaborating with community members as co-authors on all study presentations and manuscripts. To ensure frequent opportunities for formal feedback, youth and adult CABs convened quarterly. CAB members shared community perspectives and cultural insight to provide project direction and guidance to the researchers to help improve recruitment, engagement, and retention, and problem-solve cultural or implementation barriers.

The resulting Family Strengthening Intervention for Refugees (FSI-R) has the central goal of promoting family functioning to reduce risk of mental health problems among youth. Designed for refugees, by refugees, the intervention was constructed to be delivered by trained community member interventionists across ten 90-min weekly home-visiting sessions. Interventionists selected from the communities all had shared life experiences as refugees navigating the resettlement process, were often parents, and had prior experience in social services or case management but did not hold advanced mental health degrees. Each session covered different topics related to resettlement and family well-being, such as improving communication, navigating the US education system, and learning positive parenting strategies. A central part of the intervention is the development of the ‘family narrative’ whereby families reflect on their migration journey to date and choose important family events to discuss, with the interventionist highlighting the family’s unique strengths in overcoming their challenges [46]. The intervention ends with a family meeting, which brings together all members of the family—with parents leading—to reflect on their experience and goals for the future [46].

The Master’s-level FSI-R Program Manager provided daily staff support and oversaw quality improvement. Weekly supervision was provided to the interventionists by on-site clinical supervisors who also provided group supervision to interventionists two times per month [47]. The study investigators provided “super supervision” on the overall implementation and problem solving implementation barriers and a child psychiatrist provided periodic consultation. Families were offered referrals when appropriate for more intensive mental and physical health services (including substance abuse treatment) and case management.

#### FSI-R Conceptual Model and Theory of Change

**Figure 1:**
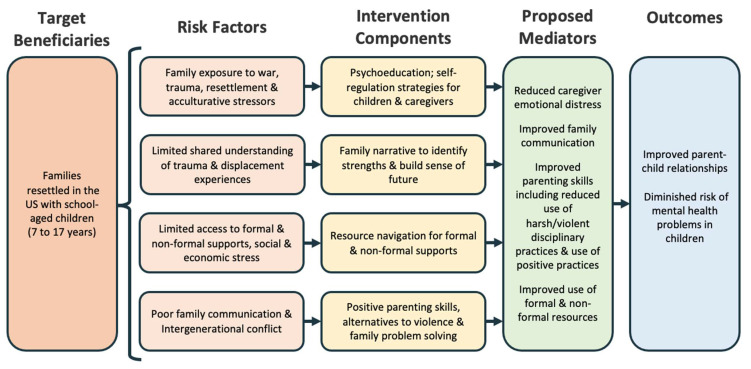
Refined conceptual model of the FSI-R.

The Family Strengthening Intervention for Refugees, which has been described in previous literature [46,47], is based on a prevention framework that aims to reduce the incidence and prevalence of mental health problems in refugee children by targeting known risk factors in the family and broader resettlement context and strengthening the coping mechanisms of children and parents (see Figure 1, which presents an updated conceptual model following on that which was published previously [46]). The intervention is informed by several complementary theoretical frameworks that inform intervention components. The FSI-R is based on developmental and ecological models of human development which view mental health problems in children and adolescents as the result of a dynamic interplay between factors in the family, community and broader society [48]. Using evidence-based components including psychoeducation, positive parenting and problem-solving skills, the model aims to strengthen key family processes including parent–child relationships, parenting skills and navigation of informal and formal resources to promote child and adolescent mental health.

The FSI-R is also informed by the stress adjustment paradigm [49] which identifies stress as resulting from an imbalance between external or internal demands and the personal and social resources to cope and manage them. To facilitate adjustment, the model includes components that aim to increase understanding of different forms of and sources of stress, particularly those common to refugee families and self-management of stress using techniques of behavioral activation, mindfulness and by strengthening social support.

The intervention also draws on the strengths-based perspective which views individuals holistically through the lens of their strengths and capacities and which are seen as important resources to be harnessed to address challenges and facilitate resilience [50]. A principal way that strengths are identified in the FSI-R is through the development of a family narrative that includes important life experiences prior to and since resettling in the U.S., challenges experienced and strengths and problem-solving capacities that the family utilized to overcome adversity. The process of developing the family’s narrative with attention to strengths and capacities is conceptualized to bring the family together to reflect on the past, present and future while promoting recognition and activation of strengths to promote functioning.

### 1.3. Study Aims

By adopting a mixed methods sequential explanatory approach [51], and examining the perspectives of both intervention participants and interventionists, the present study allows us to unpack the general pattern of pilot study outcomes which were published previously [47]. The aim of this mixed-methods analysis is to examine what factors, including characteristics of participants as well as the feasibility and acceptability of the intervention, may underlie the quantitative results of the FSI-R pilot.

## 2. Materials & Methods

The study was approved by the Institutional Review Board at the Harvard T. H. Chan School of Public Health. Adult participants provided oral consent, children under the age of 18 years and younger provided oral assent, and primary caregivers provided oral consent for their children to participate. Research Assistants (RAs) who were also hired from the resettled communities and spoke the same languages (i.e., Maay Maay or Nepali as well as English), recruited all participants via phone calls, home visits, and community events. RAs completed ethics training online in addition to intensive training and supervision from study staff.

In order to be eligible for the study, families needed to have U.S. government refugee status, at least one child ages 7–17 years, and have lived in the U.S. for at least three months. If families were experiencing a crisis such as severe psychiatric illness, ongoing legal proceedings, they were referred to a higher level of care and were not enrolled in the pilot. Eligible families were randomized into the FSI-R or care-as-usual (CAU) arms of the study, described in previous publications [47].

Families in both arms participated in quantitative child and caregiver assessments in Nepali, Maay Maay, or English, which occurred pre- and post-FSI-R delivery. The post-tests were meant to be given to families immediately after completing the intervention. Some families took longer to move through the intervention modules than others; for intervention participants, the mean time between pre- and post-test was 12.91 months, and for CAU families, it was 9.81 months. RAs were blinded to group assignment and trained to collect survey data on digital tablets. In all, 146 Somali Bantu individuals participated in quantitative data collection (103 children and 43 caregivers) as well as 111 Bhutanese individuals (49 children and 62 caregivers).

Twenty families per community in the FSI-R arm of the study were selected via a random-digits table to participate in qualitative exit interviews post-FSI-R. One child and one caregiver were asked to complete semi-structured interviews from each family, conducted in Nepali, Maay-Maay, English, or a mix of languages. Out of the 20 families per community recruited, 11 Bhutanese families and 10 Somali families participated (n = 10 Somali Bantu caregivers, n = 8 Somali Bantu children, n = 9 Bhutanese caregivers, n = 9 Bhutanese children). These interviews were conducted between January and March 2019.

Finally, four interventionists (n = 3 Bhutanese and n = 1 Somali Bantu) also participated in semi-structured interviews about their experiences delivering the FSI-R.

Family participant demographic information is presented in Table 1.

### 2.1. Measures

Data on intervention acceptability, feasibility, and family and child outcomes were collected using quantitative measures [47] and semi-structured qualitative interview guides [50], as described in prior publications.

#### 2.1.1. Quantitative Acceptability and Feasibility Measures

In order to evaluate intervention acceptability and feasibility, FSI-R participants were asked to complete an 11-item survey after completing the intervention. Items were either yes/no questions (e.g., “Would you recommend the FSI-R to a friend or neighbor?”), or scored on a scale of 0 to 2, where 0 represented dissatisfied, 1 was neither satisfied nor dissatisfied, and 2 was satisfied (e.g., “How satisfied were you with the FSI-R interventionist?”). These satisfaction questions were re-scaled into percentages, where the percent satisfied was calculated as the percentage of “2” responses.

#### 2.1.2. Quantitative Family Outcome Measures

Full descriptions of the outcome measures and adaptations made for this study have been published elsewhere [47]. Child-reported parenting practices were assessed using an adaptation of the Alabama Parenting Questionnaire (APQ) [52] developed for this study. Subscales included positive parenting (α = 0.83, 6 items, high scores representing greater positive parenting, such as whether caregivers use encouraging language (i.e., telling the child they “are doing a good job” on homework, etc.), poor monitoring (α = 0.88, 10 items, high scores representing poorer monitoring, such as whether the child “stay[s] out in the evening past the time [they] are supposed to be home”), and parental involvement (α = 0.77, 10 items, high scores representing greater involvement, such as whether caregivers “play games or do other fun things” with the child). Responses could range from 1 (“never”) to 5 (“always”), and the score on each subscale was the sum of its items. Intergenerational congruence was measured with a version of the Intergenerational Congruence in Immigrant Families Scale [53] modified for this study to include 17 items scored from 0 (“never”) to 4 (“every day”); items include, for example, “My father/mother and I agree on types of friends I have” and “My father/mother and I agree on the amount of time we spend together”. The scale was calculated as mean scores across items (child α = 0.92; caregiver α = 0.90).

#### 2.1.3. Quantitative Child Outcome Measures

Depression and anxiety were assessed with the child-report (α = 0.85) and caregiver-report (α = 0.84) versions of the 20-item Center for Epidemiologic Studies Depression Scale for Children (CESD-C). Responses ranged from 0 (“not at all”) to 3 (“a lot”) and were summed. Child-reported suicidal ideation was measured using the CESD-C supplement. Functional impairment (understanding and communicating, mobility, self-care, getting along with people, life activities, and participation in society) was assessed using the child report (α = 0.82) and caregiver-report (α = 0.86) versions of the 14-item WHO Disability Assessment Schedule for Children 2.0 (WHODAS-Child) [54]. Responses, which were summed, ranged from 0 (“no difficulty”) to 4 (“extreme difficulty/cannot do”). Child-reported trauma symptoms and traumatic events experienced were assessed using the short form of the University of California at Los Angeles (UCLA) PTSD Reaction Index [55]. Items were yes-no questions (1 = yes, 0 = no) and the scale score was the sum (α = 0.85). Finally, child-reported (α = 0.87) and caregiver-reported (α = 0.90) conduct problems were measured using the 32-item externalizing subscale of the Achenbach Youth Self Report (YSR) and Child Behavior Checklist (CBCL) [56]. Responses could range from 0 (“not true”) to 2 (“very/often true”) and were summed to create the scales.

#### 2.1.4. Qualitative Semi-Structured Interviews

Exit interviews with caregivers and children covered several key topics, including: general family-level experiences with the intervention; feasibility of family participation; satisfaction with various intervention aspects; how families may have been affected by the intervention; use of skills learned in FSI-R; topics found most important; perceptions of family functioning and relationship dynamics post intervention; relationships with other community members post intervention; experiences with the interventionist; perceptions of FSI-R prior to participation; and ways to improve the future iterations of the intervention.

Interviews with interventionists covered related topics, including: general experiences delivering the intervention; perception of the intervention’s impact on participating families; intervention components found to be most useful to families; variations in needs across participating families; techniques used to build rapport with families; differences across family member participation and engagement; strategies for engaging all family members during intervention sessions; perceived satisfaction of participating families; personal satisfaction with the intervention; experience training and being supervised as an interventionist; and experiences connecting families to additional services.

### 2.2. Analysis Strategy

Our analysis used an explanatory sequential mixed methods design, wherein we discuss the ways and extent to which the quantitative results are explained by the qualitative interview data [51]. This strategy allows for a product in which the whole (i.e., the mixed methods analysis) is greater than the sum of its parts (i.e., the quantitative data plus the qualitative data).

#### 2.2.1. Quantitative Analysis Strategy

The details of the quantitative statistical analyses have been published elsewhere [47], and used mixed effects models to estimate the effect of the FSI-R on change in quantitative measures, separately for the Bhutanese community and for the Somali Bantu community, from pre- to post-test. A chained equations method in Stata 15 accounted for missing data [57]. All models controlled for participant sex, date of birth, number of people in the household, years the participant had lived in the U.S., city (as families lived in one of two metropolitan areas), time between pre-test and post-test, and a sum of trauma events experienced by the participant (measured with the UCLA PTSD Reaction Index Trauma History Profile [55]). The coefficients are point estimates comparing the pre- to post-test change in outcome scores of the intervention participants versus CAU participants.

#### 2.2.2. Qualitative Analytic Strategy

A detailed account of our qualitative analysis processes has also been published elsewhere [50]. We employed Grounded Theory to analyze the interview transcripts and detailed field notes [58]. A highly iterative and team-based process was followed to improve validity and interpretation [59]. First and second authors served as primary coders and conducted deep readings of each transcript and made “open codes”, discussing their impressions with the authorship to allow for an iterative meaning-making process [60]. After refining codebooks, they met to negotiate meaning across sets of categories and codes and consolidated them into a final codebook. The coders then re-coded a subset of the interviews, discussing coding applications until they met consensus. The larger authorship team then employed axial coding to discuss connections within categories and themes. Finally, authors who were members of each refugee community offered final feedback to ensure trustworthiness [59]. All qualitative analyses were carried out using MAXQDA 2018 [61].

## 3. Results

### 3.1. Family Outcomes

FSI-R family outcomes are displayed in Table 2. Caregivers, children, and interventionists reported seeing meaningful changes in their families due to the intervention, especially with regard to communication and spending time together, and to a lesser degree, in caregiver discipline practices, as well.

Somali Bantu children and caregivers noted that after the intervention, they began to take time to be with one another, especially amongst siblings. As a Somali Bantu mother, aged 38, noted, before the intervention: *“children were not coming out from their room. Recently, they sat in the living room to share how school was and share daily living activities.”* They also noted increased communication, including in terms of caregivers listening to their children. One Somali Bantu 55-year-old father explained, *“We never talk priority for my kids, always working working. But [the intervention] give me small lesson[s] that I also need to listen to them about their thought[s].”*

A Somali Bantu interventionist also reflected on similar changes he saw in the families with whom he worked, stating,

*“Through the intervention, changes that I’ve seen was like starting from the first module, through to the end I see changes where before they were not like connecting or listening to each other, but now, what they hear from me or learn from me they made a big improvement and learned from me through the modules.”*

Somali Bantu families also made changes in their parenting practices (although this topic presented some acceptability issues, as discussed in subsequent sections). A 37-year-old mother stated that the most important topic for her family was parenting, stating, *“I was punished before by my parent with a stick. Here in the US, [we are] not allowed to punish with [a] stick or belt, only talking to them politely.”* She went on to explain, *“Before the intervention, as a parent I used to yell at my kids all the time. Now [we have] better communication, I talk to them as an adult now, in [a] respectful way.”* Likewise, one Somali Bantu interventionist did see changes in caregivers’ thinking and behaviors around discipline:

*“During the modules like for example module 4 …parenting strategies, we see disciplining the kids was need[ing] to be, like, taught…before, they didn’t even know anything about that but right now, you know, they have made some new changes about what they have learned through tools from me.”*

Bhutanese families and interventionists also described important improvements in communication and time spent as a family. As one interventionist described, *“I also noticed…that the parents are more involved in asking what is going on in the school systems, how their sons, or how their students are making progress or not.”*

A 40-year-old mother reflected, *“We do home family meeting and gathering, and talk about school, and felt more awareness and importance of talking with kids, felt closeness, know[ing] kids’ needs, and sharing. Very helpful.”*

However, interventionists also noted that they did not see the same degree of change in all families. Bhutanese interventionists believed that caregivers with higher education levels were better primed to implement the parenting practices they were taught in the intervention. One interventionist explained:

*“With one family... Probably due to their educational status being a bit higher, both caregivers were educated. I got [a] very positive response from them. They said they used to have family discussions in some ways, but they realized they could do it differently and effectively, as discussed in the intervention.”*

Another Bhutanese interventionist explained that, *“Every family has their own comprehension level. The families with a very low comprehension level, or illiterate families were also there.” He found that these families with “low comprehension levels”* would tell him it was not necessary to implement practices from the FSI-R:

*“It will be different in different families. Some families are in good condition. These families take what I taught and suggest during intervention very positively; therefore, we can see positive impacts on these families, whereas some families do not even care, they are good until the intervention session exists, but after the session is over, they do not follow what was taught or said during the intervention session. I have continuously followed up with those families, but then they respond [that] they are okay! And their lifestyle or [their] relationship does not does not require any changes....For this, I can see I found impact in families 50–50.”*

In this small-sample pilot, the differential impact between families well-positioned to receive the intervention and families facing more barriers to behavior change could have muted the quantitative improvements.

### 3.2. Child Outcomes

FSI-R child mental health outcomes are described in Table 3. Bhutanese families tended to remark on child mental health improvements more often than Somali Bantu families. Several Bhutanese families spoke about children seeming less anxious due to the intervention. A 12-year-old girl explained,

*“Before this intervention, I wouldn’t tell anything that happened at school to my parents because I got really worried, cause I used to get bullied. Now, I tell my parents and they help me a lot. And my sisters, they’re there for me too. And I actually tell a lot of stuff to my parents about school, because that makes me more comfortable going to school and learning.”*

A Bhutanese mother from another family, age 44, noted similar changes in her son.

*“It’s been different. Like my child, if I have to say, is of scared type. But he’s not scared these days. He comes to me and says what needs to be done. It’s been good. It’s been going well in family too. I also understand more now, how children need to be loved. I experienced like that and it’s been good for children as well.”*

In addition, a Bhutanese boy, aged 16, from another family explained, *“Now… I’m not shy anymore … I can help my family without being scared of them or what they think about me.”* He also noted, *“the Family Intervention has…made my family happier…more engaging with each other.”*

Similar to the qualitative accounts, Bhutanese children were observed to have significantly reduced depression (β = −9.20, *p* = 0.04) following the FSI-R intervention compared to families in usual care.

Bhutanese children also had a reduction in conduct problems (β = −0.92, *p* = 0.01). Though this was not explicitly mentioned by participants in the qualitative data, a Bhutanese mother, age 40, described that they *“felt more importance of the family. Kids [were] also much [more] obedient and working together. Going time for each other, avoiding electronics devices, closeness with each other, limiting the electronics and focusing more on studies.”*

In contrast, when asked what changes they saw in their families, Somali Bantu caregivers and children who participated in the intervention, as well as the Somali Bantu interventionist, did not bring up child mental health.

Because mental health problems are often stigmatized in diverse refugee communities, the FSI-R places more emphasis on psychoeducation around resettlement stressors, the impact of displacement on wellbeing and various approaches for coping and promoting resilience in families. Once more rapport is built, later modules of the FSI-R do emphasize the importance of health promotion and accessing services, including mental health services where relevant. One Bhutanese interventionist explained how the topic of mental health was extremely new for families:

*“I know our community have very little knowledge about mental health, and people really doesn’t want to talk about it, and people really doesn’t want to seek mental health help and mental health providers. So I think that piece of mental health information in that module [of the FSI-R] was very helpful, and were having healthy conversation about those things…. In terms of impact, I did saw people started using mental health services.”*

However, Bhutanese children had measurable mental health improvements even amidst this stigma.

### 3.3. Intervention Acceptability

#### 3.3.1. Overall Satisfaction

Both the quantitative and qualitative data indicate that the intervention was overall highly acceptable to both communities. Nuances in the quantitative outcomes, however, can be further explained through the qualitative findings (Table 4). When asked directly about satisfaction, 85.7% of the Bhutanese community indicated that they were satisfied (the remaining 14.3% were neither satisfied nor dissatisfied) compared to 76.9% the Somali Bantu community being satisfied. However, when measuring satisfaction indirectly, via willingness to participate again and willing to recommend to a neighbor or friend, the Somali Bantu responded with 100% satisfaction, while the Bhutanese community came in lower at 64.3% and 85.7%, respectively. One possibility for the discrepancy between general satisfaction and more specific indicators of satisfaction among Bhutanese is the cultural emphasis placed on politeness and the desire to not offend an interventionist whom they know from the community. A Bhutanese interventionist noted, *“I have felt that they [participants] might have been dissatisfied [though]...they did not say openly that they were dissatisfied.”* This possibility notwithstanding, much of the qualitative data supported the majority of both communities indicating high levels of satisfaction and continuing to practice what they learned in the FSI-R.

Exit interviews with participants triangulated our quantitative finding that more Bhutanese reported that they were continuing to practice skills learned in the FSI-R compared to those in the Somali Bantu community. A 40-year-old Bhutanese mother described *“doing [family meetings] often… encouraged to do so from the intervention,”* and a 17-year-old Bhutanese girl from another family said they do theirs *“every Sunday.”*

Even though Somali Bantu families said the intervention was, for example “fun,” “very helpful,” and that their “kids loved it,” and 100% of caregivers said they would recommend the FSI-R to a friend or neighbor, some caregivers also described it being difficult to use what was learned in the intervention due to limited time. A 32-year-old mother explained, *“who has the time? I am working all day,”* while a 40-year-old Somali Bantu mother said, *“I like asking my child a question about his day at school, talk[ing] to them. [But] some of the things that [the interventionist] telling us need[s] time, and I don’t have time.”* It is evident here how acceptability is intimately related to feasibility; the quantitative discrepancies between acceptability measures within each community may relate to whether or not participants felt it was possible to integrate what was learned in the intervention into their daily lives.

#### 3.3.2. Meeting the Participants’ Needs

Fifty-four percent of Somali Bantu participants reported that the intervention met their needs, whereas the Bhutanese community’s report was higher, at 78.6% (Table 4). Members of both communities brought up the desire for more practical skills, including English language learning, applying to jobs, financial literacy, and building connections across the community.

One 42-year-old Somali Bantu mother described: *“I was so excited, but at the end not happy… If you give us ways to save money, learn English, job training, and many other things that other offices do, it help[s] the community.”* The same participant also shared that the intervention *“talk[ed] about things that we already know,”* and that she would have preferred to learn information that was new to her. It is possible that this family already had strong parenting and communication skills and was looking to gain other practical skills.

The diverse needs of families posed difficulties for interventionists, who sometimes felt like they were limited in the help that they could offer families. A Bhutanese interventionist mentioned feeling like he did not always have the tools needed to assist, saying in some cases, *“the resources to address these needs are not known to us either.”* He went on to explain:

*“[R]esources regarding healthcare, information [about] different types of benefits available for families, educational resources, information on job opportunities; these sorts of information on available resources are handy to us. But, if some families come up with complex problem[s] then, we might not have information on available resources which would help address those issues...”*

#### 3.3.3. Interventionists

In both communities, satisfaction with the interventionist was 100% (Table 5), which participants attribute to their existing relationships or familiarity with them. Some had known their interventionists from their time in refugee camps, while others simply appreciated that the interventionists were members of their community and that they *“talk[ed] to me in my language.”* As a 42-year-old Somali Bantu mother described, *“I don’t see anyone else in the community that will be able to do that job.”* It was clear from members of both communities that the high levels of satisfaction were due to the inherent trust they felt in someone who shared their experiences, language, and culture. These sentiments were echoed by interventionists themselves, as one Bhutanese interventionist shared, *“[The families] were very familiar with me, which made the process easier for them as well as for me… I didn’t have to go through any such challenges to build up the relationship with the[m]...If I had to work with strangers or new people then it would have taken more time as I would not have been familiar with their culture.”*

#### 3.3.4. Content, Exercises, and Information Gained

Satisfaction with the contents of the intervention, exercises, and information gained were high for both communities (Table 5). Interviews with participants revealed that both communities particularly appreciated the modules covering the US education system, parenting strategies, and ways to spend time as a family. Though differences were minor, Somali Bantu were slightly less satisfied with the content and exercises of the FSI-R compared to the Bhutanese community, though Somali Bantu satisfaction with both was still high at 76.9%. Several Somali Bantu caregivers cited struggling with the FSI-R’s content regarding child discipline, as it was different from what was accepted in their culture. A mother, age 37, explained, *“When it come[s] to disciplin[ing] children, it was uncomfortable for me. I thought if I tell you about what I do you would report me*.” Another mother, also 37, shared that she *“personally like [learning] parenting strategies, but [there is] still no proper way of disciplining the children. Talking to children politely, saying please to your child- I didn’t like that.”* This is explored further in Section 3.3.

Another possible explanation for slightly lower satisfaction among Somali Bantu compared to Bhutanese was the language barrier across generations within the family, an issue frequently brought up by Somali Bantu caregivers and children. In several cases, mothers only spoke Maay Maay while their children spoke only English. Several Somali Bantu caregivers reported language barriers as a frustrating aspect of the intervention, sharing: *“the family meeting went well, but most of the time my own children were speaking English,” “my children speak English but we don’t,”* and *“some of my kids don’t speak Maay Maay, so it was very difficult for me to initiate communication with them.”* Bhutanese participants reported very different language experiences within the family in the context of the intervention, as one child remarked that *“English is very important [to my caregiver],”* and therefore speaking English was encouraged although several Bhutanese families also expressed valuing their children’s efforts to learn Nepali and using it at home.

### 3.4. Intervention Feasibility

Feasibility of the FSI was assessed quantitatively by asking caregivers about their satisfaction with the intervention length and their ability to get through each session in an efficient manner (Table 6). Bhutanese caregivers rated their satisfaction slightly higher than Somali Bantu caregivers, yet both were above 75% satisfaction. Interviews with caregivers, children, and interventionists alike reveal possible explanations for less than 100% satisfaction, many of which indicate difficulty with gathering family members at the same time and place to participate. Several interventionists described difficulty navigating caregiver’s schedules, especially around employment and other commitments that conflicted with intervention meeting times. Regarding the length of the intervention, some reported that it was too lengthy, while many Somali Bantu caregivers in particular indicated that they could have used *“more meeting session[s];” “more time to meet with the family;”* or that *“each family should get longer.”*

## 4. Discussion

This mixed methods study adds nuance and depth to previous research which (1) indicated that the FSI-R pilot was generally acceptable and feasible with Bhutanese and Somali Bantu communities in New England and (2) found positive patterns in improved parenting skills and child mental health, with the need to have the effectiveness outcomes confirmed in a fully powered trial. Interviews with intervention participants reinforced these findings (including community member interventionists contributing to acceptability and feasibility and children’s improved mental health), as well as suggested several plausible explanations for the potential patterns of differences in certain outcomes by the community. Qualitative interviews suggest that families with varying levels of education and social status may have responded differently to FSI-R. In addition, unique feasibility challenges for the Somali Bantu participants, such as large family sizes and language barriers between caregivers and children, will need greater attention in future iterations of the model.

Children who received the intervention demonstrated improvements in parent-reported depression and child-reported PTSD symptoms. Participants talked about how children were more comfortable opening up to their caregivers after the FSI-R. Indeed, previous literature indicates that refugees, such as previous studies with Bhutanese refugees suffering from mental health problems “keep their problems to themselves and try to work things out on their own” [62], so the FSI-R’s approach to strengthening communication and connectedness is highly relevant for this community.

Regarding family outcomes, many interventionists and participants spoke about important changes that the FSI-R brought about. In Somali Bantu families, both children and caregivers reflected on how the FSI-R brought siblings together and strengthened their relationships. This is consistent with prior research on resettled Somali Bantu families, which found that sibling bonds strengthened after resettlement as their shared experiences led siblings to rely on one another for support, including in the midst of strained caregiver-child relationships [63]. Sibling relationships were not assessed in this quantitative pilot but were often reflected upon in participant interviews, and therefore could be an important mediator or moderator of child mental health outcomes to examine in future studies.

Some unique challenges facing each community were illuminated by this mixed methods study. For example, participating Somali Bantu families had many children—almost three times as many as Bhutanese families, on average [47]—and both participants and interventionists spoke about how difficult it was to find space in everyone’s competing schedules to hold FSI-R sessions with the whole family at once.

Language barriers between Somali Bantu caregivers and children also created a significant feasibility challenge to implementing the FSI-R, while also serving as a risk factor for poor family functioning. Because Somali Bantu families resettled several years before the Bhutanese families, many of the Somali Bantu youth were born in the United States, and they often did not learn their native language Maay Maay. Prior literature describes not only a language barrier between caregivers and children, but also a cultural divide, with caregivers sometimes frustrated with their children for not retaining their cultural and religious practices as they assimilate to American life [63].

Although both groups showed improvements in ratings of PTSD and depression in children, more mental health improvements were demonstrated in the Bhutanese in this small pilot sample. Similarly, Bhutanese interventionists said that they witnessed meaningful changes in family functioning due to the intervention, though not necessarily in all families. Quantitative changes were not detected on the scale used to assess family functioning which may be attributed to the small pilot sample or measurement issues. Consistent with the transtheoretical model of behavioral change, wherein individuals are theorized as having different levels of readiness to change their behavior [64], Bhutanese interventionists said that some caregivers were ready and willing to adopt new practices such as family meetings or positive parenting approaches, while others were not. They stated that more “educated” families were better positioned to adopt behavior changes. This is interesting in light of literature that has found that amongst resettled Bhutanese adults, illiteracy in the Nepali language and lower educational attainment are risk factors for mental health problems [29,30]. Future research should investigate the links between education, literacy, mental health, and parenting in this population. Although the Somali Bantu interventionist did not mention seeing differential effects among families (and the fact that only one Somali Bantu interventionist was able to participate in interviews limits the claims that can be made about patterns of change observed in families from that community), prior research has found that Somali Bantu community members were often unable to access education in their homeland and during displacement which led to various struggles in their resettled life [63].

Health and mental health interventions aimed at facilitating behavior changes amongst diverse populations, especially resettled and immigrant groups, must navigate an inevitable tension that arises from introducing new ideas from the dominant culture to replace individuals’ existing practices. This can be even more fraught in the context of parenting and families, where culture and homeland are intimately involved in influencing familial relationships. If done without care, behavior change interventions can serve as little more than an intrusive vehicle of assimilation. For these reasons, the FSI-R relied carefully on CBPR principles at every stage of development and implementation. Interventionists, who came from participants’ cultural communities, were carefully trained on navigating the more controversial topics, such as discipline and positive parenting, and did not teach caregivers that particular behaviors were “wrong.” Rather, they introduced parenting strategies that may have been unfamiliar to them that they could draw upon if they found them useful in navigating their new lives in the United States.

The findings of this study point to the importance of tailoring interventions to meet participants where they are. Indeed, the overall findings suggest high levels of feasibility, acceptability among families engaged in the intervention. These findings very likely reflect the fact that the intervention was developed in close partnership with refugee communities, gaining input from community stakeholders along the way to craft a model that would be appropriate and responsive to the needs of families. Taken as a whole, this suggests that family-based mental health services can be useful for resettled communities. Qualitative insights from this study also shed light on future adaptations to family-based mental health services including strengthening attention to practical needs and supports (e.g., English language learning, employment) as part of an overall approach to service delivery. As the refugee crisis continues to grow globally, the ability to quickly adapt interventions to be culturally appropriate for different refugee populations becomes increasingly important. An intervention done “for refugees, by refugees,” in line with CBPR principles, has tremendous potential as an acceptable and feasible approach to quickly pivoting to address the needs of diverse refugee groups in need of support.

### Limitations

This study also has important limitations. While interventionist interviews suggested there may be a differential impact on Bhutanese caregivers by educational attainment, the quantitative portion of this pilot study, only containing 20 Bhutanese intervention families, is too small to untangle whether the same dynamic played out in the quantitative data. As the FSI-R is adapted and evaluated in more refugee communities, it will be important to examine such treatment moderators in better powered samples, while also investigating treatment mediators (i.e., mechanisms of change). Another validity issue pertains to social desirability bias wherein, due to cultural norms, Bhutanese adults were very reluctant to say things that could be perceived as negative, despite the efforts of the interviewers (whom all spoke Nepali) in this study to probe for both positive and negative experiences.

Another limitation is that the qualitative interview protocol had more questions about acceptability and feasibility than it did questions about specific mental health and family functioning outcomes. For this reason, interview data did not contain information that could explain every construct measured in the quantitative survey. Future research should investigate both qualitative and quantitative data on intervention mediators such as parental involvement, positive parenting, communication and conflict management along with potential moderators such as educational level, literacy and family size on outcomes such as intervention effects on symptoms of depression, anxiety, traumatic stress reactions.

## 5. Conclusions

The FSI-R is a promising family-based preventative intervention developed using CBPR approaches to promote functioning and child mental health in resettled refugee families. This randomized pilot trial with Somali Bantu and Bhutanese refugees in New England showed that the FSI-R reduced child depression symptoms and PTSD in refugee children. Interventionists described generally positive acceptability and feasibility driven by the experience with peer providers who knew their language and culture. In some cases, potential differences were hypothesized such as seeing more “educated” families adopt new parenting practices more readily than other families or language barriers limiting parent–child communication. Such dynamics should be investigated in future research along with how the integration of such programs into broader community initiatives may also address some of the unmet needs for tangible inputs such as language, savings and cultural initiatives for families in addition to family-based mental health promotion. Future research should continue to examine the mediators and moderators of FSI-R impact so that the intervention can continue to be adapted for the unique needs of a diverse array of refugee families.

## Figures and Tables

**Table 1:** Demographic characteristics of family participants in quantitative and qualitative portions of the study.

		Individuals, n	Female, n (%)	Age, M (Range)	Years in U.S., M (Range)
Quantitative study participants (n = 257)	Somali Bantu children	103	61 (59%)	14.6 (8–22)	8 (8–15)
	Somali Bantu caregivers	43	34 (79%)	41.8 (28–70)	13.3 (12–22)
	Bhutanese children	49	26 (53%)	14.4 (8–18)	4.0 (1–8)
	Bhutanese caregivers	62	32 (52%)	41 (27–66)	4.3 (1–10)
Qualitative sub-study participants (intervention families only) (n = 36)	Somali Bantu children	8	4 (50%)	14.5 (11–17)	12.7 (11–15)
	Somali Bantu caregivers	10	9 (90%)	40.1 (32–52)	13.3 (12–15)
	Bhutanese children	9	3 (33%)	15.7 (12–18)	4.7 (1–8)
	Bhutanese caregivers	9	4 (44%)	46.1 (34–60)	4.6 (1–7)

**Table 2:** Joint display table: Integration of quantitative (point estimates comparing pre- to post-test change of the intervention versus CAU participants) and qualitative (interview data) about family outcomes.

Quantitative Results			Qualitative Results	Interpretation
Construct	SB (β)	B (β)		
Poor monitoring ^†^	0.01	−0.01	“We do practice the skills or information we learned in the intervention, [like] family meeting, spending more playtime with the kids, meeting with school teachers regularly.”—*Somali Bantu mother, age 37* “We are interacting more with each other, keeping [in the] loop with school, and also continue doing family talking time together. I started calling school to know about the informations.”—*Bhutanese mother, age 40* “Some families…take what I taught …very positively; therefore, we can see positive impacts on these families, whereas some families do not even care…after the session is over they do not follow what was taught or said during the intervention session.…For this, I can see I found impact in families 50–50.”*—Bhutanese interventionist* “Probably due to their educational status being a bit higher…I got [a] very positive response from them…they realized they could do [family discussions] differently and effectively, as discussed in the intervention.”*—Bhutanese interventionist*	Families reported meaningful impacts in communication and spending time together. These were important changes according to the participants and may also have contributed to the findings observed on intergenerational congruence as reported by parents and children. Interventionists perceived differential impacts based on family education level and literacy. Such differential outcomes could have muted overall quantitative results in a small-sample pilot.
Parental involvement ^†^	−5.41 *	−0.07		
Positive parenting ^†^	0.06	0.11		
Intergenerational congruence ^†^	−0.06	0.04		
Intergenerational congruence ^‡^	−0.46	−0.04		

SB = Somali Bantu, B = Bhutanese. ^†^ Child-reported; ^‡^ Caregiver-reported; * *p* < 0.05

**Table 3:** Joint display table: Integration of quantitative (point estimates comparing pre- to post-test change of the intervention versus CAU participants) and qualitative (interview data) about child mental health outcomes.

Quantitative Results			Qualitative Results	Interpretation
Construct	SB (β)	B (β)		
Depression/anxiety ^†^	0.18	−0.07	“Our community ha[s] very little knowledge about mental health and people really doesn’t want to talk about it and people really doesn’t want to seek mental health help…so I think that piece of mental health information in that module was very helpful, and were having healthy conversation about those things.… In terms of impact, I did saw people started using mental health services.” —*Bhutanese interventionist* “My child…is of scared type. But he’s not scared these days. He comes to me and says what needs to be done.”—*Bhutanese mother, age 44*	Mental health was a very stigmatized topic for families, many of whom were learning about the importance of mental health promotion and how to access mental health services for the first time. Bhutanese children had reduced adult-reported depression and anxiety, and many Bhutanese families spoke of their children’s reduced anxiety in interviews. Somali families, on the other hand, did not comment on child mental health. The intervention’s sample size was too small to detect any impacts on child mental health across measures.
Depression/anxiety ^‡^	−0.06	−9.20 *		
Functional impairment ^†^	−0.02	−0.15		
Functional impairment ^‡^	−0.01	0.13		
Conduct problems ^†^	0.17	−0.34		
Conduct problems ^‡^	1.48 ***	−0.92 *		
Suicidal ideation ^†^	0.55 *	−0.79		
Trauma symptoms ^†^	−0.30	−0.28		

SB = Somali Bantu, B = Bhutanese. ^†^ Child-reported; ^‡^ Caregiver-reported; * *p* < 0.05, *** *p* < 0.001.

**Table 4:** Joint display table: Integration of quantitative (percent of caregivers reporting construct) and qualitative (interview data) about acceptability of the intervention.

Quantitative Results			Qualitative Results	Interpretation
Question	SB (%)	B (%)		
Satisfied in general	76.9%	85.7%	“[You should] add more families [to the intervention] and more times.”—*Somali Bantu mother, age 47* “Others also need this [intervention].” —*Bhutanese boy, age 16* “Very satisfied. It was fun, especially when we all together discussing about our family.”—*Somali Bantu mother, age 37* “When you first come…my thought was there is a program starting in the community for all family to participate but it was just one-on-one meeting and family meeting asking questions. I didn’t like it. People need actual help. Start a family program…like you remember the money saving we did before? Like that.”—*Somali Bantu mother, age 42* “When we explained [to] them our boundaries, what we could do and what we could not do for them, they felt a bit uncomfortable.”—*Bhutanese interventionist* “Resources regarding healthcare,… education,…job opportunities; these sorts of…resources are handy [available] to us. But, if some families come up with complex problem then, we might not have information on available resources…such as violence, sexual assault, children out of school, mental health.”—*Bhutanese interventionist*	Families expressed general satisfaction with the intervention and the intervention appeared to be acceptable overall. Families have many other needs, e.g., financial, that were not met by a talk-based intervention alone. Although interventionists were able to connect participants with community resources, interventionists sometimes felt they lacked the information they needed for complex problems, and some families needed more than referrals (such as intensive case management); interventionists sometimes felt the help needed exceeded their ability to deliver.
Willing to participate again	100%	64.3%		
Would recommend to a neighbor/friend	100%	85.7%		
Continues to practice skills/knowledge from FSI-R	61.5%	100%		
FSI-R met the participants’ needs	53.8%	78.6%		

SB = Somali Bantu, B = Bhutanese.

**Table 5:** Joint display table: Integration of quantitative (percent of caregivers reporting construct) and qualitative (interview data) about acceptability of the intervention (cont.).

Quantitative Results			Qualitative Results	Interpretation
Question	SB (%)	B (%)		
Satisfied with the interventionist	100%	100%	“Still I get phone calls from some of the families, [laughs] they miss us so much.”—*Bhutanese interventionist* “It was more comfortable working with [the interventionist] because I knew him [before the intervention].”—*Bhutanese boy, age 18*	Families were very happy with their interventionists across the board. Hiring fellow community members as interventionists was an effective strategy for intervention acceptability.
Satisfied with content	76.9%	92.9%	“We are satisfied with the intervention because we learned a lot that we didn’t know, like parenting strategies, education system in the U.S., family meeting.”—*Somali Bantu mother, age 37* “We have learned many things from this program. Like…things we should do to help kids in their school…that we should talk to their teachers…and how they are doing in school…we learned these things that we didn’t really know before.” —*Bhutanese father, age 65* “Too much unnecessary information. Who doesn’t know parenting? Once you get a child, your mind and body automatically start thinking like a parent.”—*Somali Bantu mother, age 42*	Respondents generally reported satisfaction with the skills and knowledge gained, especially around the U.S. school system. Still, there were some barriers to acceptability, especially among Somali Bantu caregivers. In particular, non-violent discipline practices, though important to introduce, may not be fully embraced in a ten-session intervention.
Satisfied with exercises	76.9%	100%		
Satisfied with information gained	100%	100%		

SB = Somali Bantu, B = Bhutanese.

**Table 6:** Joint display table: Integration of quantitative (percent of caregivers reporting construct) and qualitative (interview data) about feasibility of the intervention.

Quantitative Results			Qualitative Results	Interpretation
Question	SB (%)	B (%)		
Satisfied with length	76.9%	92.9%	“Most of the time there was, ‘Okay Mrs./Mr. So-and-So is not here today, so you want to come back another time?’ You see one time a father is not home, but the mother says…, ‘Okay today my husband is not here and my son is not there as well, so you want to just reschedule again’… If the father is missing or the mother is missing then the intervention wasn’t fully delivered the way it is supposed to be.”—*Somali Bantu interventionist*	It was difficult to bring all the members of a family together in one place for sessions due to their busy schedules, especially for Somali Bantu families, which tended to be larger.
Satisfied with how family was able to get through each session	76.9%	100%		

SB = Somali Bantu, B = Bhutanese.

## Data Availability

The data used in this study are not public at this time.
